# Fabrication of a lattice structure with periodic open pores through three-dimensional printing for bone ingrowth

**DOI:** 10.1038/s41598-022-22292-z

**Published:** 2022-10-14

**Authors:** Jong Woong Park, Hyenmin Park, June Hyuk Kim, Hong Moule Kim, Chang Hyoung Yoo, Hyun Guy Kang

**Affiliations:** 1 Orthopaedic Oncology Clinic, Center for Rare Cancers, National Cancer Center, Goyang-si, Gyeonggi-do Republic of Korea; 2 Surgical Oncology branch, Division of Clinical Research, National Cancer Center, Goyang-si, Gyeonggi-do Republic of Korea; 3 HANA AMT Company, Cheongju, Republic of Korea

**Keywords:** Surgical oncology, Health care, Biomedical engineering

## Abstract

Lattice structures for implants can be printed using metal three-dimensional (3D)-printing and used as a porous microstructures to enhance bone ingrowth as orthopedic implants. However, designs and 3D-printed products can vary. Thus, we aimed to investigate whether targeted pores can be consistently obtained despite printing errors. The cube-shaped specimen was printed with one side 15 mm long and a full lattice with a dode-thin structure of 1.15, 1.5, and 2.0 mm made using selective laser melting. Beam compensation was applied, increasing it until the vector was lost. For each specimen, the actual unit size and strut thickness were measured 50 times. Pore size was calculated from unit size and strut thickness, and porosity was determined from the specimen’s weight. The actual average pore sizes for 1.15, 1.5, and 2.0 mm outputs were 257.9, 406.2, and 633.6 μm, and volume porosity was 62, 70, and 80%, respectively. No strut breakage or gross deformation was observed in any 3D-printed specimens, and the pores were uniformly fabricated with < 10% standard deviation. The actual micrometer-scaled printed structures were significantly different to the design, but this error was not random. Although the accuracy was low, precision was high for pore cells, so reproducibility was confirmed.

## Introduction

**Figure 1:**
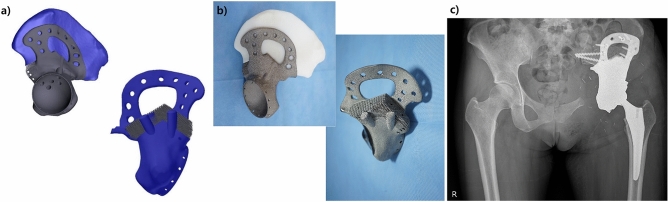
Pelvic implant manufactured by metal 3D printing for a 35-year-old woman who received surgery for Ewing sarcoma of the left pelvis. (**a**) Designs and (**b**) photographs revealed that the pelvic implant has both solid and lattice structures. (**c**) A postoperative plain radiograph of the applied pelvic implant.

Three-dimensional (3D)-printed implants, which are mainly used in orthopedic oncology to reconstruct bone defects after bone tumor removal, can be customized for patients^1–5^ (Fig. 1). Currently, metal 3D-printed orthopedic implants are manufactured using the powder bed fusion method; however, the electron-beam melting (EBM) method^2,6–8^ has a faster fabrication speed, produces less pore defects, and has greater thermodynamical stability than the selective laser melting (SLM) method because it prints at high temperatures and high beam energy under a vacuum^5,9^.

Implants must have both body strength and bone growth potential at the junction to the host bone. Considering practical use, a solid and periodic cellular structure, which is called the lattice structure, should have a clear division of roles in orthopedic implants and be properly mixed in each customized implant (Fig. 1). The mechanical properties of 3D-printed Ti6Al4V solid structure have been shown to be suitable for orthopedic implants compared to those of traditionally fabricated solid structures^10^. However, lattice structures used for securing biocompatibility and bone ingrowth have poor mechanical properties and microstructural weakness^11^, so load bearing can be difficult to achieve with them^1–5^. The bone-inducing ability of the lattice structure with Ti6Al4V has been previously reported in animal experiments and human studies^12–15^. For example, Fig. 1 presents the case of a woman who received limb salvage surgery for the left pelvis using a 3D-printed custom-made implant in our institution (details of the fabriction conditions for 3D-printed human implants were described in Supplementary 1). The pelvic implant was designed using the patient's images, which were obtained by computed tomography (CT) and magnetic resonance imaging. The implant was made with Ti6Al4V and fabricated using an EBM type 3D-printer. To enhance bone ingrowth, the junction where the bones and implants make contact was designed to have a lattice structure. Regarding the patient presented in Fig. 1, this study protocol was approved by the institutional review board of National Cancer Center (NCC2017-0129). The present study was conducted according to the principles of the Declaration of Helsinki. Written informed consent was obtained from a participant prior to inclusion in the study.

Targeted microstructures with open pores to enhance bone ingrowth used in orthopedics have an average pore size of 300–600 μm, with a volume porosity of 70%^16–19^. Previous studies have reported that titanium scaffolds with open pores with appropriate pore size and volume porosity demonstrate an osteoconduction effect^20–24^. Both EBM and SLM types of 3D-printing have been applied for cellular lattice structures, and SLM was found to fabricate finer lattice than EBM^16^. However, even using the SLM method, differences were noted between the design and actual products, especially in micrometer-scaled structure fabrication^25–28^. In our experience, reliable pore size is decreased by 675 μm using the EBM method, and lattice structures with open pores of 675 μm are commonly used for customized 3D-printed Ti6Al4V implants. However, the lattice structure was designed as a dode-thin structure with unit size of 2.0 mm by Magics software (Magics RP 22, Materialise, Leuven, Belgium) and a pore size of 790 μm. From a technical point of view, developing a structure in which the design and 3D-product match as much as possible is important; however, if the error is predictable, it may be possible to select and use a lattice structure with an appropriate unit size despite errors, in the clinical field.

This study aimed to quantify fabrication errors between designs and actual 3D products with various pore sizes, and to determine if printing errors are random or reproducible, that is, whether targeted pores can be consistently obtained despite printing errors.

## Methods

### Specimen fabrication

**Figure 2:**
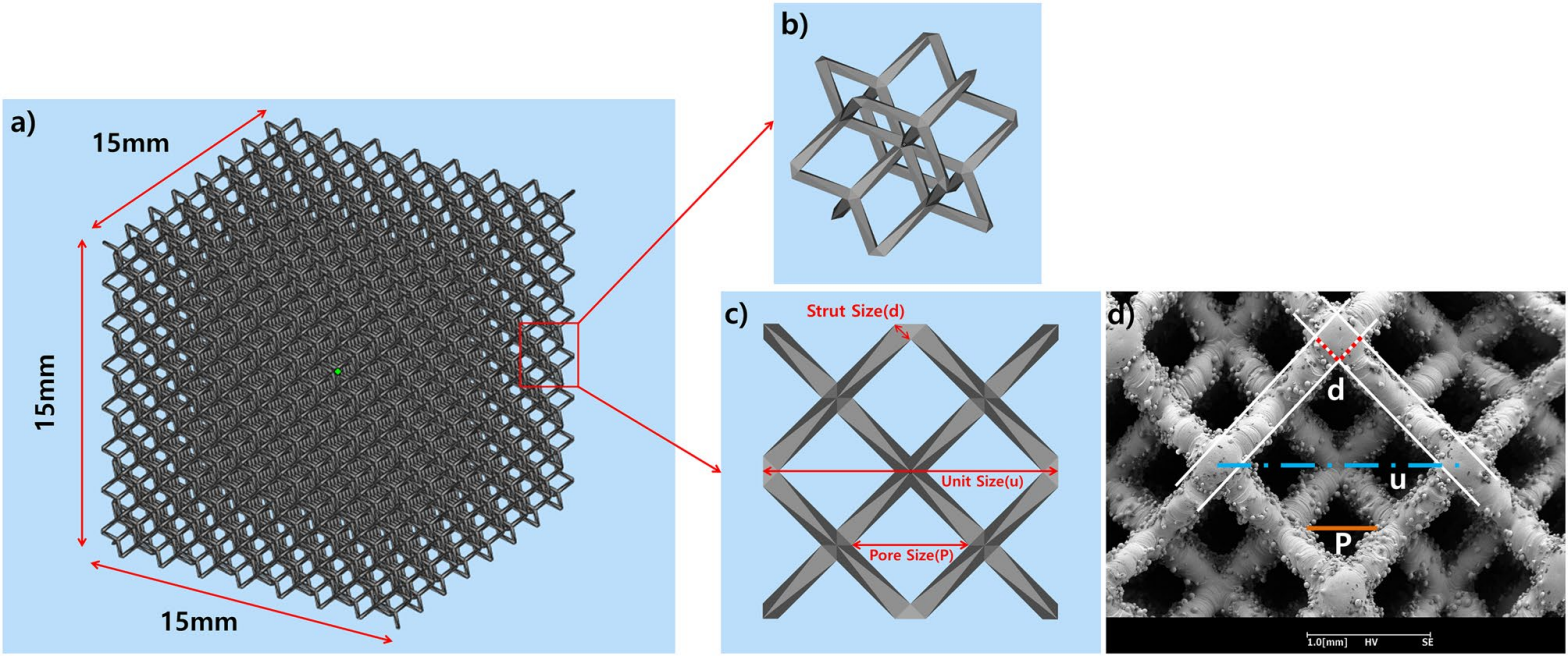
Design and measurement of the lattice structure. (**a**) An overview of the lattice structure (dode) and a unit cell design (**b**) in oblique view and (**c**) top view. (**d**) An image of the scanning electron microscope with auxiliary lines for measurement.

**Figure 3:**
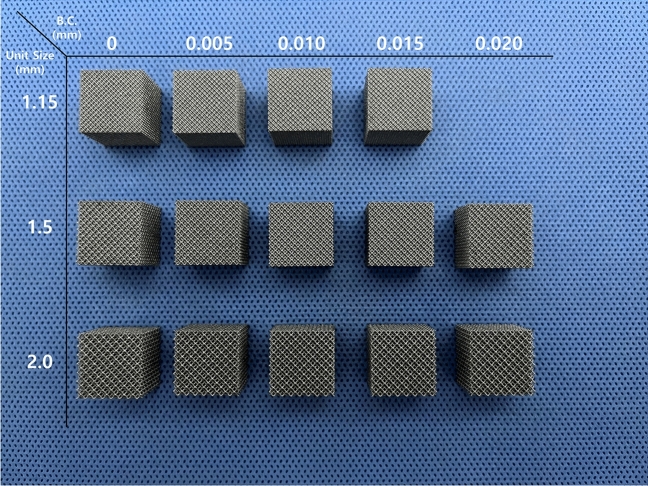
Specimens printed by the selective laser melting method.

Specimens with a lattice structure were printed in the shape of a cube with a side length of 15 mm. The lattices had a dode-thin lattice structure, designed using Magics software (Magics RP 22, Materialise, Leuven, Belgium) with unit sizes of 1.15 mm (A), 1.5 mm (B), and 2.0 mm (C). Specimens were printed using an SLM method printer Dpert M200 (DAEGUNTECH, Changwon, South Korea) and Ti6Al4V ELI powder (Joy Company, Cheongju, South Korea) with a particle size between 15 and 53 μm. The basic process conditions of the SLM method were as follows: laser power (110 W), laser scan velocity (1,050 mm/s), laser diameter (0.05 mm), and layer thickness (30 μm). The beam compensation value was applied and sequentially increased by 5-μm intervals from 0 to printing failure due to vector loss. The beam compensation was a correction value of reducing the design by a few micrometers to prevent over-sizing due to the melting pool of the laser beam in the printing margin. In microstructure printing, the strut of the lattice structure is a few micrometers, so printing vectors were lost when the beam compensation value exceeded a certain value, resulting in printing errors^26^ (Figs. 2 and 3). All parameters except beam compensation and postprocessing procedures, such as sandblasting, were the same as those for 3D-printed human implant production conditions.

### Measurement of porosity

The strut and unit sizes were measured using a scanning electron microscope (SEM) EM-30AX (COXEM, Daejeon, South Korea). For each specimen, 50 units of lattice were scanned and measured. All measurements were performed independently by two engineers (HMP and CHY) and averaged.

Pore size was calculated as follows (Fig. 2d):

$$P=u\times \frac{1}{2}-d\times \sqrt{2}$$

where P = pore size; u = unit size; d = strut size.

The density of the solid part was 4.37 g ${\mathrm{cm}}^{-3}$ for Ti6Al4V, and the densities of the lattice specimens were determined by measuring the mass and volume of specimens. The mass was measured using an electronic scale (CBL-220H, CAS Corporation, USA) with an accuracy of 0.01 g. The volume of the cube specimen shape was measured using a Vernier caliper (CD-20AX, Mitutoyo, Japan) with an accuracy of 0.01 mm, based on the lengths of each of the three sides. Volume porosity was obtained by the following equation:

$${\text{p}}_{actual} = \left( {1 - \frac{{\rho_{{{\text{Lattice}}}} }}{{\rho_{Ti} }}} \right) \times { }100(\% )$$

### Statistical analysis

Continuous variables were compared using the independent-samples t-test. To examine the beam compensation effects on pore size, one-way analysis of variance (ANOVA) was performed. Statistical analysis was performed using the SPSS v. 21.0 software (IBM Inc., Armonk, New York). All reported P values were two-tailed, and significance was set at < 0.05.

## Results

In total, 14 specimens were printed. By increasing beam compensation, vector loss occurred at 20 μm for specimen A (unit size, 1.15 mm) and 25 μm for specimens B and C (unit size, 1.5 and 2.0 mm, respectively) (Fig. 3). No strut breakage or gross deformations were observed in any of the 3D-printed specimens.

**Figure 4:**
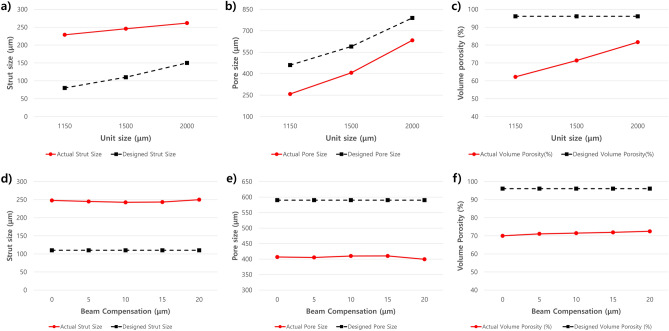
Graphs for the actual and designed measurements. (**a**) An increase in strut thickness of ≥ 100 µm was observed for all unit sizes, and due to this, (**b**) the pore size decreased compared with the design. (**c**) As the unit size increased, the volume porosity also gradually increased. By beam compensation, (**d**, **e**, **f**) no trend of actual measurements was observed within small variations, such as in specimens with a unit size of 1500 μm. (**a, b, d, e, f)** The number of errors in strut thickness and pore size was constant by unit size and beam compensation. (**c**) For volume porosity, with increase in unit size there was a decrease in the number of units and struts in the 1.5-mm cube specimens; global errors of porosity also decreased. Overall, the effects of unit size design and beam compensation were minimal, and all errors stemmed from the constant increase in strut width due to the melting pool at the edge of the struts.

The mean pore sizes were 257.8 ± 23.9, 406.2 ± 17.4, and 633.6 ± 26.3 μm for specimens A, B, and C, respectively. For each specimen, the standard deviations of actual pore sizes were < 10.0%, 4.7%, and 5.9% for specimens A, B, and C, respectively. The means of volume porosity were 62.3, 71.4, and 81.6% for specimens A, B, and C, respectively. For all specimens, designs and actual measurement values were significantly different (all *p* < 0.01). In particular, actual pore sizes were all significantly smaller than the original designs (all *p* < 0.01). The means of differences between design and measurements for pore size were − 202.1, − 183.8, and − 156.4 for specimens A, B, and C, respectively (Table 1, Fig. 4).

**Table 1:** Differences between designed and actual size of specimens.

No	Unit size (μm)	B C (μm)	Strut size				Pore size				Volume porosity			
			Designed value (μm)	Actual value (μm)	Difference value (μm)	Difference (%)	Designed value (μm)	Actual value (μm)	Difference value (μm)	Difference (%)	Designed value (%)	Actual value (%)	Difference value (μm)	Difference (%)
A00	1159.8	0	80	220.3	140.3	175.4	460	268.4	− 191.6	− 41.66	96.1	61.9	− 34.2	− 35.6
A05	1158.5	5	80	227.9	147.9	184.9	460	256.9	− 203.1	− 44.15	96.1	63.7	− 32.4	− 33.7
A10	1170.1	10	80	231.2	151.2	189.0	460	258.1	− 201.9	− 43.9	96.1	62.4	− 33.7	− 35.1
A15	1162.8	15	80	235.8	155.8	194.7	460	248.0	− 212.0	− 46.1	96.1	61.0	− 35.1	− 36.5
Avg: A	1162.8		80	228.8	148.8	186.0		257.9	− 202.1	− 43.95		62.3	− 33.8	− 35.2
B00	1514.6	0	110	247.9	137.9	125.3	590	406.7	− 183.3	− 31.06	96.1	70.0	− 26.1	− 27.2
B05	1503.2	5	110	245.0	135	122.7	590	405.2	− 184.8	− 31.32	96.1	71.1	− 25.0	− 26.0
B10	1505.8	10	110	242.6	132.6	120.5	590	409.9	− 180.1	− 30.52	96.1	71.5	− 24.6	− 25.6
B15	1508.6	15	110	243.4	133.4	121.3	590	410.0	− 180.0	− 30.5	96.1	71.9	− 24.2	− 25.2
B20	1505.6	20	110	249.9	139.9	127.2	590	399.3	− 190.7	− 32.31	96.1	72.5	− 23.6	− 24.5
Avg: B	1507.6			245.8	135.8	123.4		406.2	− 183.8	− 31.14		71.4	− 24.7	− 25.7
C00	2004.2	0	150	266.4	116.4	77.6	790	625.4	− 164.6	− 20.83	96.1	80.2	− 15.9	− 16.5
C05	2001.9	5	150	269.5	119.5	79.7	790	619.8	− 170.2	− 21.54	96.1	81.7	− 14.4	− 14.9
C10	2010.3	10	150	256.8	106.8	71.2	790	642.0	− 148.0	− 18.73	96.1	82.9	− 13.2	− 13.7
C15	2003.5	15	150	256.8	106.8	71.2	790	638.6	− 151.4	− 19.16	96.1	81.6	− 14.5	− 15.0
C20	2015.5	20	150	258.4	108.4	72.3	790	642.3	− 147.7	− 18.7	96.1	81.7	− 14.4	− 14.9
Avg: C	2007.1			261.6	111.6	74.4		633.6	− 156.4	− 19.79		81.6	− 14.5	− 15.0

The pore size was calculated from unit size and strut thickness. The means of unit size were 1162.8 ± 17.5, 1507.6 ± 12.5, and 2007.1 ± 19.0 μm for specimens A (unit size, 1.15 mm), B (unit size, 1.5 mm) and C (unit size, 2.0 mm), respectively. Negligible global enlargement or reduction in unit size was observed. Meanwhile, the means of strut thickness were 228.8 ± 16.4, 245.8 ± 11.9, and 261.6 ± 16.7 μm for specimens A (designed strut 80 μm), B (designed strut 110 μm), and C (designed strut 150 μm), respectively. Therefore, most of the pore size reduction may have been due to the increased strut thickness (Table 1, Fig. 4).

The effects of beam compensation for pore size in each specimen had significant differences (ANOVA, specimen A, *p* < 0.01; specimen B, *p* = 0.01; specimen C, *p* < 0.01). Maximal differences in pore size by beam compensation were 20.4, 10.7, and 22.5 for specimens A, B, and C, respectively. However, the pore size changes by beam compensation were random, without exhibiting any clear tendency.

## Discussion

Metal 3D printing with Ti6Al4V is used for orthopedic implant fabrication in clinical practice; solid and lattice structures are mixed in single orthopedic implants. The main purpose of using lattice structures is not mechanical support but osteoconduction. Open pores that have a 300–600 µm diameter and 70% volume porosity are reported to have appropriate values for enhancing cell penetration and bone ingrowth^16–19^. However, when micrometer-scaled structures are fabricated by metal 3D-printing, differences in design and 3D-printed products have been reported, with differences of > 100 µm in struts of open cells^28–30^. In this study, the reproducibility of SLM micrometer-scaled structures was confirmed, and predictable printing errors were identified. Despite the differences between design and actual 3D-products, a targeted open porous structure for bone ingrowth was obtained uniformly.

The choice of lattice structure may be worth considering, depending on the type of orthopedic implant, although it is not important in most cases, including customized megaprostheses in limb salvage surgery. The mechanical properties of lattice structures are dependent on unit cell conformation^15,31^. The yield strength of the various lattice structures reportedly ranges within tens of MPa and a similar level of strength; hence, using lattice structures alone as orthopedic implants may not be suitable^11,15,31^. Except for some spacers for small bone defects that induce bone regeneration and internal bone bridging, the role of lattice structures in orthopedic implants is limited to providing osteoconduction and mechanical stability, depending on the solid structure. Biocompatibility and osteoconduction are related to appropriate pore size and independent of unit cell feature^15,32^. Therefore, rather than comparing the mechanical performance of lattice structures, the question of whether a uniform structure with appropriate pore size is well-printed without strut breakage, and whether residual metal powder is sufficiently removed after sandblasting is much more important clinically. Herein, a single lattice design was investigated without comparing various structures, and it was judged to be suitable for clinical use in terms of reproducibility of unit cells, with no breakage of struts or closing of pores, and minimal residual powders.

The strut output was approximately 100 μm thicker than the design, which was consistent with the results of the previous literatures^28–30^. Regardless of the strut design, the output > 200 μm is related to the width of the melt pool^28^. In this study, the actual struts in the lattice structure were thicker than those in the design, and the amount of added thickness was independent of beam compensation in SLM (Table 1). To control the melting pool width, the beam compensation was set differently, although the effect was random without any tendency, and negligible compared to the standard deviation of strut thickness measurements. Laser power and scan velocity are related to melting pool size, but adjustment of these parameters could lead to uncontrolled porosity in the inside struts^33^. Minimizing the difference between the design and actual output by reducing the melting pool size is important in terms of fabrication optimization. However, from a clinical perspective, having a certain degree of predictable difference from the design will not have a serious negative impact as long as the lattice structure has the appropriate pore size without building failure.

**Figure 5:**
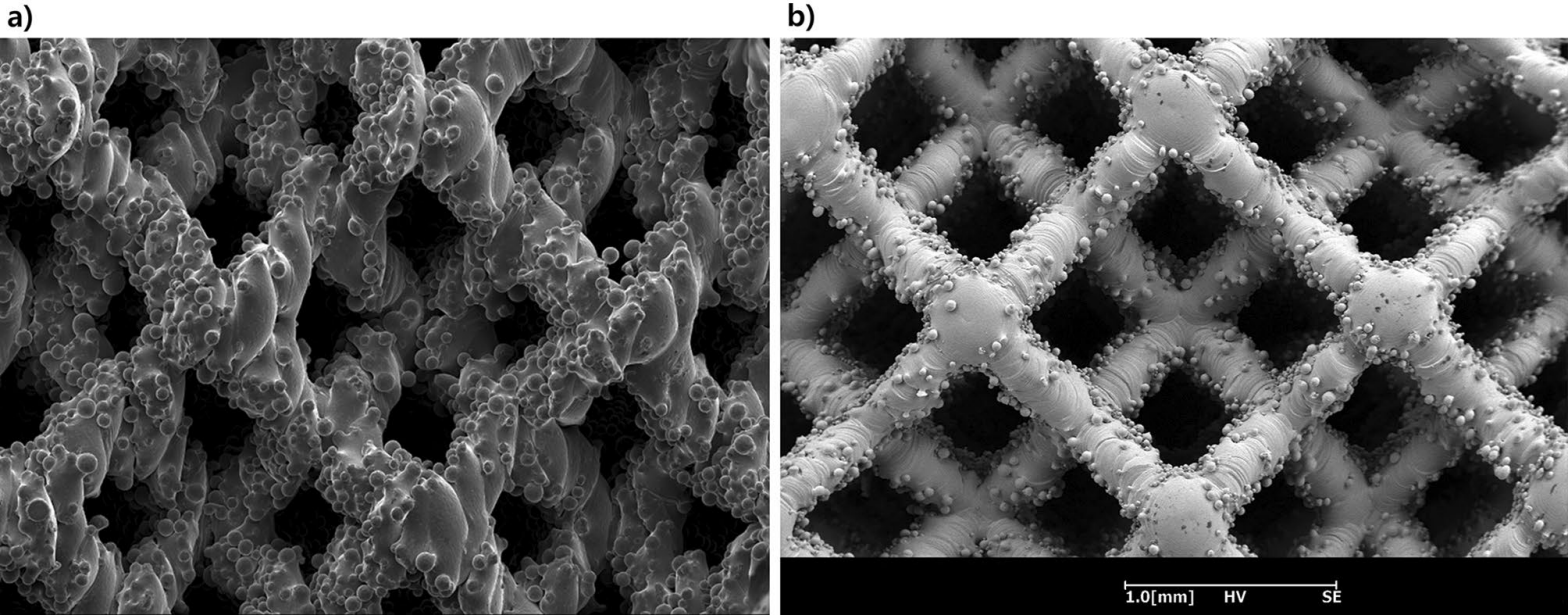
Comparison of strut surfaces. Images of the scanning electron microscope for the same lattice structure with a unit size of 2.0 mm, which was fabricated (**a**) by electron-beam machining and (**b**) selective laser melting, revealed sintered unmelted powders on the surfaces.

Zhao et al. reported that the size of the partially melted powder on the surface in the SLM method was significantly smaller than that of the EBM method^9^. Sintered unmelted powders on a surface and lattice structure for use as orthopedic implants for a fractures have also been reported^11^. To compare the surfaces of struts that made by SLM and EBM methods, the same lattices with a 2.0-mm unit size were fabricated and examined with SEM (Fig. 5). The SLM specimen had less sintered unmelted powders on the strut surfaces than the EBM specimen. Thus, the SLM method can print finer pores than those printed via the EBM method.

CT offers many advantages in the inspection of 3D-printed products and processes; however, there is a limitation in micrometer-scaled measurements. The inspection method for geometry or internal defects of 3D-printed products in real-time during printing or after fabrication has not been standardized yet^34–36^. Nevertheless, CT is a good inspection tool for non-destructive testing^37^; internal pores and porosity are detected without destruction, but micrometer-scaled measurements are not reproducible. For example, Du et al. reported that pore detection and distribution were acceptable, but porosity values (range 0.012–0.03%) and maximal pore size (0.178–0.85 mm) varied among 10 different laboratories, even when using the same micro-CT analysis protocols^34^. Sources of deviation included scanning and image analysis errors. In the present study, 14 specimens of a cube composed of lattice structures were scanned using micro-CT with a pixel size of 10 μm and upper and lower grey thresholds were 60 and 255, respectively. All specimens were non-destructively analyzed by micro-CT and confirmed to have no strut breakage. However, micrometer-scaled measurements were not reliable. For example, for specimen B (lattice unit size, 1.5 mm), the average strut thickness measurements were 290.4, 289.2, 290.3, 291.5, and 291.5 μm, with beam compensation of 0, 5, 10, 15, and 20 μm, respectively. The strut thickness measurements obtained by micro-CT scans were greater than those from SEM scans by approximately 45 μm. Pore size and porosity (%) of the cubes obtained by micro-CT scans were subsequently lower than those from SEM scans. The calculated mass (g), which was obtained by multiplying the volume of all struts in each cubic specimens (calculated with the imaging software), by the density (4.43 g/cm^3^) of Ti6Al4V ELI, was larger than the actual mass of the specimens; therefore, it was concluded that the struts in micro-CT were over-measured. In other words, although micro-CT was useful for non-destructive evaluation of internal defects such as strut breakage, it was unreliable as a micrometer-scale measuring tool.

This study had several limitations. First, the specimens were examined in as-built status. This method was appropriate for observing errors due to printing itself, but the pore sizes might have changed due to deformation after heat treatment. Second, the targeted open pore, which has the best osteoconductivity, was well implemented by SLM; however, an additional in-vivo study is required to confirm this. Last, measurement errors may have occurred. To overcome this problem, each type of lattice was randomly measured 50 times and averaged by two independent engineers.

In metal 3D printing of micrometer-scaled structures, a reproducible error was observed between the design and actual product. Nevertheless, in the SLM method, producing an optimal porous structure with a pore size of 300–600 μm and a porosity of 70% was possible, considering repeated errors.

## Supplementary Information

Supplementary Information.

## Data Availability

The datasets generated during and/or analysed during the current study are available from the corresponding author on reasonable request.
