# Supplementary material for: Fabrication of a lattice structure with periodic open pores through three-dimensional printing for bone ingrowth
Source: Sci Rep. 2022 Oct 14;12:17223. doi: 10.1038/s41598-022-22292-z (PMC9568544; doi:10.1038/s41598-022-22292-z)
Supplement: Supplementary file 1 — Supplementary Information. [file 41598_2022_22292_MOESM1_ESM.docx]

**Supplementary 1.** Design and fabrication of 3D-printed customized Ti6Al4V implants for human limb salvage surgery

The design process for customized 3D-printed implants required close communication between orthopedic oncologists and engineers. Computerized tomography (CT) and magnetic resonance imaging (MRI) scans with a thin section thickness of 1–2 mm were used in the design process. All medical images were stored in Digital Imaging and Communications in Medicine (DICOM) format. A graphical 3D model was created using CT and MRI, and virtual resection were performed using MIMICS (Interactive Medical Image Control System; Materialise; Leuven, Belgium). The shape of the implant body was obtained by applying the virtual cutting planes to produce a mirror image of the contralateral unaffected bone. The implants included both lattice and solid structures to enhance bone ingrowth and to support mechanical strength. The dode-thin mesh structure was applied as a lattice structure using Magics 22 (Materialise; Leuven, Belgium). After 3D-printed implant fabrication, a polishing process was completed to prevent abrasion or adhesion to a major neurovascular bundle. For implant fabrication, 3D printing was performed with medical-grade titanium (Ti6Al4V-ELI Per ASTM 136) using an electron beam melting (EBM) 3D printer (ARCAM A1, GE additive, Boston, United States). The MEDYSSEY Company (Jecheon, Korea) custom-made implant was certified by the Ministry of Food and Drug Safety. The maximum build size was 200×200×180 mm^3^, with an accuracy of 0.2 mm.
